# Do mental health consumers want to improve their long‐term disease risk behaviours? A survey of over 2000 psychiatric inpatients

**DOI:** 10.1111/inm.12411

**Published:** 2017-12-02

**Authors:** Kate Bartlem, Jacqueline Bailey, Alexandra Metse, Ashley Asara, Paula Wye, Richard Clancy, John Wiggers, Jenny Bowman

**Affiliations:** ^1^ School of Psychology Faculty of Science and Information Technology University of Newcastle Callaghan New South Wales Australia; ^2^ Clinical Research Centre Hunter Medical Research Institute Newcastle New South Wales Australia; ^3^ Population Health, Hunter New England Local Health District Wallsend Health Services Newcastle New South Wales Australia; ^4^ School of Nursing and Midwifery Faculty of Health and Medicine University of Newcastle Callaghan New South Wales Australia; ^5^ Centre for Translational Neuroscience and Mental Health Hunter New England Mental Health Mater Hospital Newcastle New South Wales Australia; ^6^ School of Medicine and Public Health Faculty of Health and Medicine University of Newcastle Callaghan New South Wales Australia

**Keywords:** mental health services, long‐term disease, smoking; nutrition, alcohol consumption, physical activity

## Abstract

Policies and clinical guidelines acknowledge the role mental health services have in addressing the physical health of individuals with a mental illness; however, little research has explored interest in reducing health risk behaviours or the acceptability of receiving support to reduce such risks among psychiatric inpatients. This study estimated the prevalence of four long‐term disease risk behaviours (tobacco smoking, hazardous alcohol consumption, inadequate fruit and/or vegetable consumption, and inadequate physical activity); patient interest in reducing these risks; and acceptability of being provided care to do so during a psychiatric inpatient stay. A cross‐sectional survey was undertaken with 2075 inpatients from four inpatient psychiatric facilities in one health district in Australia (October 2012–April 2014). Prevalence of risk behaviours ranged from 50.2% (inadequate physical activity) to 94.8% (inadequate fruit and/or vegetable consumption). The majority of respondents (88.4%) had more than one risk behaviour, and most were seriously considering improving their risk behaviours (47.6% to 65.3%). The majority (80.4%) agreed that it would be acceptable to be provided support and advice to change such behaviours during their psychiatric inpatient stay. Some diagnoses were associated with smoking and hazardous alcohol consumption, interest in reducing alcohol consumption and increasing fruit and/or vegetable consumption, and acceptability of receiving advice and support. The findings reinforce the need and opportunity for psychiatric inpatient facilities to address the long‐term disease risk behaviours of their patients.

## Introduction

The life expectancy of people across the broad spectrum of mental illness is substantially lower than that of the general population (Chang *et al*. 2011; Laursen *et al*. 2013; Lawrence *et al*. 2013; Olfson *et al*. 2015; Walker *et al*. 2015). Data from Australia, the United Kingdom, and the United States estimate this life expectancy gap to be between 10 and 30 years, depending upon setting, psychiatric diagnosis, and severity of illness (Chang *et al*. 2011; Laursen *et al*. 2013; Lawrence *et al*. 2013; Olfson *et al*. 2015; Walker *et al*. 2015). Up to 80% of excess mortality in this population is estimated to be attributed to long‐term physical diseases such as cardiovascular diseases, respiratory diseases, and cancers (Brown *et al*. 2010; Druss *et al*. 2011; Lawrence *et al*. 2013). The elevated burden of long‐term disease among people with a mental illness is contributed to substantially by an increased likelihood of engaging in key modifiable risk behaviours, including tobacco smoking, hazardous alcohol consumption, inadequate nutrition, and inadequate physical activity (Callaghan *et al*. 2014; Lim *et al*. 2012; Stanley & Laugharne 2014).

The increased likelihood of individuals with a mental illness engaging in these key modifiable risk behaviours has been consistently reported in community‐based studies, including, but not limited to samples of people with severe mental illness (Bartlem *et al*. 2015a; Dipasquale *et al*. 2013; Janney *et al*. 2014; Kilbourne *et al*. 2009; Morgan *et al*. 2012). In a recent Australian survey, the prevalence of risk was considerably higher for clients of community mental health services compared to clients of generalist community health services for tobacco smoking (51% vs 13%), hazardous alcohol consumption (43% vs 22%), inadequate nutrition (87% vs 81%), and inadequate physical activity (47% vs 28%) (Bartlem *et al*. 2015a; McElwaine *et al*. 2013). The evidence regarding differences in the prevalence of long‐term disease and associated health risk behaviours as a function of diagnosis is equivocal. Some studies have identified differences in risk prevalence between psychiatric diagnostic groups (Kilbourne *et al*. 2009; Kilian *et al*. 2006), with such findings being suggested to reflect poorer physical health with increasing severity of mental illness, particularly for schizophrenia or psychosis (van Hasselt *et al*. 2015). In contrast, clients attending community mental health services reported a similar prevalence of risk behaviours across all psychiatric diagnoses (Bartlem *et al*. 2015a), and recent global research suggests that all diagnoses, not just severe mental illness, are associated with increased risk of long‐term disease (Scott *et al*. 2016). These findings indicate that strategies to address these behaviours for all people with a mental health condition, regardless of diagnosis, are required, and more research is required to determine how to best tailor health behaviour interventions for people with mental illnesses.

Mental health services have been identified as having a key role in providing care to reduce the preventable long‐term disease burden in this population (de Hert *et al*. 2011; Lambert *et al*. 2017), as reflected in the development of various national and international clinical guidelines for mental health services regarding the management and care of long‐term disease risk behaviours (Expert Reference Group to the Council of Australian Governments Working Group of Mental Health Reform 2013; National Institute for Health and Care Excellence 2014, 2015; New South Wales Mental Health Commission 2014). However, consistent evidence demonstrates that care provision in accordance with these guidelines is suboptimal in mental health services (Bartlem *et al*. 2014, 2015b; Chwastiak *et al*. 2013; Greening 2005; Prochaska *et al*. 2004; Stockings *et al*. 2015). To date, most of this research has been conducted in outpatient or community mental health services (Bartlem *et al*. 2014, 2015b; Chwastiak *et al*. 2013; Greening 2005). The limited research involving psychiatric inpatient facilities indicates that these settings are also prone to suboptimal provision of care for health risk behaviours (Prochaska *et al*. 2004; Stockings *et al*. 2015). For example, just 36% of Australian inpatient smokers reported receiving advice to quit, while 20% reported receiving best practice nicotine dependence treatment (corresponding with clinical practice guidelines) (Stockings *et al*. 2015). Inpatient settings may prove particularly difficult for the provision of care for health risk behaviours; however, little research has explored the barriers specific to providing such care in inpatient facilities. A small body of research, primarily related to smoking cessation care, suggests limited training and education, a lack of guidelines and organization support, and clinician attitudes may contribute to suboptimal care (Howard & Gamble 2011; Ratschen *et al*. 2009; Robson *et al*. 2013; Wye *et al*. 2010). Understanding the clinician and patient barriers to provision of care in these settings is essential to developing appropriate interventions to improve behaviours associated with long‐term disease.

Mental health clinicians have reported concerns regarding their patients’ level of interest in changing health behaviours, and acceptability towards being provided support to address such behaviours, as barriers to providing risk reduction care (Chwastiak *et al*. 2013; Johnson & Fry 2013; Johnson *et al*. 2009; Robson *et al*. 2013; Wye *et al*. 2010). For instance, 69% of nurse unit managers from Australian psychiatric inpatient facilities report their patients have no interest in quitting smoking (Wye *et al*. 2010), while 30% of inpatient and community mental health nurses in the UK report their patients are not motivated to exercise (Robson *et al*. 2013). Several studies, however, report that such perceptions do not accurately reflect the interests of patients, demonstrating that they are interested in modifying their health risk behaviours, and are receptive to receiving care for health behaviours from mental healthcare providers. (Bartlem *et al*. 2015a; Buhagiar *et al*. 2011; Filia *et al*. 2011; Ussher *et al*. 2007).

To the authors’ knowledge, only one study has examined interest in modifying long‐term disease health risk behaviours among mental health inpatients (Prochaska *et al*. 2014). The study, undertaken in the United States, involved mental health inpatient smokers and found a variable level of patient interest in improving a range of health risk behaviours: tobacco smoking 23%, fruit and vegetable consumption 46%, physical activity 51%, and binge alcohol consumption 57% (Prochaska *et al*. 2004). To our knowledge, no studies have reported mental health inpatient acceptability of being provided care in the inpatient setting to address their long‐term disease health risk behaviours.

The aims of this study were to (i) determine the prevalence of four major long‐term disease health risk behaviours (smoking, hazardous alcohol consumption, inadequate fruit and/or vegetable consumption, inadequate physical activity) in adult psychiatric inpatients; (ii) determine adult psychiatric inpatients’ interest in improving health risk behaviours; and (iii) determine adult psychiatric inpatients’ perceptions of the acceptability of being provided advice and support for behaviour change during a psychiatric inpatient stay. Differences between psychiatric diagnostic groups were examined.

## Methods

### Design and setting

A cross‐sectional survey was undertaken with inpatients from four public adult inpatient psychiatric facilities in one regional local health district in New South Wales, Australia. Six acute clinical units (20–25 beds each) were utilized across the four facilities, one of which provided specialized drug and alcohol services to patients with a comorbid psychiatric disorder. Psychiatric emergency care short‐stay units, and units specializing in care for older persons were excluded. The study was undertaken in the context of a larger randomized controlled trial evaluating the efficacy of an integrated smoking cessation intervention (Metse *et al*. 2014). Ethical approval was obtained for the larger project from the Hunter New England Human Research Ethics committee, reference no.: 11/12/14/4.02, and the University of Newcastle Human Research Ethics Committee, reference no.: H‐2012‐0061. The reporting of this study conforms to the STROBE statement (von Elm *et al*. 2007).

### Participants and recruitment

Throughout the study period (October 2012–April 2014), research staff approached senior clinicians on a daily basis at each of the four facilities to identify eligible patients. Eligibility included being admitted to the facility during the study period, being 18 years of age or more, and identified by a senior clinician as being well enough to participate.

### Data collection procedures

Eligible patients were approached by research staff independent of the inpatient facility and asked to participate in the survey. Surveys were administered in a quiet area of the unit as a face‐to‐face interview by the research staff and took ~15 min to complete. Electronic medical records were used to attain clinical and demographic data for each participant. Summary of clinical and demographic information for patients who did not consent or were not approached was obtained in aggregate form from the facility electronic medical record system.

### Measures

#### Patient characteristics

Clinical and demographic information, collected via electronic medical records, included the following: age, gender, marital status, primary psychiatric diagnosis, identification as an Aboriginal and/or Torres Strait Islander person, and length of stay (days).

#### Health behaviour risk status

Participants reported their engagement in four health risk behaviours prior to admission to the unit. Six questions were developed around definitions of risk according to Australian national guidelines (Department of Health and Aged Care 1999; Ministerial Council on Drug Strategy 2004; National Health and Medical Research Council 2003, 2009) and have been previously used with clients of community mental health services (Bartlem *et al*. 2015a).

Participants reported, during the month prior to admission: whether they smoked any tobacco products (daily, weekly, less than weekly, ex‐smoker, never smoker); number of fruit (0; 1; 2; 3; 4; 5+; do not know; refused) serves and vegetables (0; 1; 2; 3; 4; 5+; do not know; refused) serves they usually ate each day; and number of days per week they usually did 30 minutes or more of physical activity (0; 1; 2; 3; 4; 5+; do not know; refused). Participants reported, in the last year: how frequently they consumed a drink containing alcohol (never, monthly, or less, 2–4 times a month, 2–3 times per week, 4 or more times a week); and for those who had consumed alcohol, the number of drinks containing alcohol they consumed during a usual drinking day (1 or 2; 3 or 4; 5 or 6; 7–9; 10 or more).

Health behaviour risk status was defined in line with Australian national guidelines: any smoking (daily, weekly, less than weekly) (Ministerial Council on Drug Strategy 2004); consumption of more than two standard alcoholic drinks on a usual drinking day (National Health and Medical Research Council 2009); consuming less than two serves of fruit and/or less than five serves of vegetables per day (National Health and Medical Research Council 2003); or not engaging in at least 30 min of physical activity for at least 5 days a week (Department of Health and Aged Care 1999).

#### Interest in changing health risk behaviours, and acceptability of clinical staff providing risk reduction advice

For each of the four risk behaviours, participants reported whether they were seriously considering making any positive changes (yes, no, do not know). To reduce response burden, single‐item measures of interest in change were used. Such measures have been previously used in behaviour change research (Haukkala *et al*. 2000; Luszczynska *et al*. 2007; Marques‐Vidal *et al*. 2011; Stockings *et al*. 2013) and have been demonstrated to predict behaviour change attempts (Stockings *et al*. 2013), and successful behaviour change (Sciamanna *et al*. 2000) as accurately as measures containing multiple items. To determine acceptability towards clinical staff providing risk reduction advice during their inpatient stay, participants were provided the following statement ‘*It would be acceptable for the staff looking after me whilst in the hospital to give advice and support to help with any health behaviours I would like to change’* and asked to respond on a Likert scale (strongly disagree, disagree, unsure, agree, strongly agree).

### Statistical analysis

Data were analysed using SAS analysis package (SAS, v9.4; SAS Australia, Lane Cove, NSW, Australia). Condensed response variables were created for age, marital status, and length of stay (see Table 1). Risk variables (yes, no/do not know) were created from risk status responses for each of the four health risk behaviours. Responses to fruit and vegetable consumption items were combined to create an overall nutrition risk variable (reflecting inadequate fruit and/or inadequate vegetable consumption). Participants who responded ‘do not know’ to any of the ‘interest in changing health risk behaviour’ items were considered, on a conservative basis, to not be interested in changing. Descriptive statistics were used to examine patient characteristics, the prevalence of risk for each of the four behaviours (overall and by diagnostic category), the prevalence of being at risk for multiple behaviours (overall), the interest in improving health behaviours for participants identified to be at risk (overall and by diagnostic category), and the acceptability of being provided advice and support for behaviour change (overall and by diagnostic category).

**Table 1:** Demographic comparison of participants, nonconsenters and partial completers, and not approached

	Participants (*n* = 2075)		Nonconsenters (*n* = 237)		Not approached (*n* = 1311)	
	Mean	SD	Mean	SD	Mean	SD
Age (years), mean	41.5	14.1	44.7	15.4	39.8	17.1
Length of stay (days), mean	15.8	20.6	24.7	64.9	12.4	62.1
	%	*n*	%	*n*	%	*n*
Male	55.8	1157/2075	52.3	124/237	60.0	786/1311
*Marital status* a						
Married/de facto	24.8	512/2063	19.4	45/232	26.0	337/1298
Single, separated, widowed, divorced	75.2	1551/2063	80.6	187/232	74.0	961/1298
Identifies as Aboriginal and/or Torres Strait Islander	11.5	238/2063	11.5	27/234	12.8	167/1300
*Primary diagnosis*						
Anxiety and stress related	9.0	187/2075	4.2	10/237	20.3	266/1311
Mood disorders	31.5	654/2075	25.3	60/237	23.1	303/1311
Other	3.7	77/2075	3.8	9/237	4.1	54/1311
Personality disorders	14.0	290/2075	11.4	27/237	17.2	225/1311
Schizophrenia, related psychosis	25.5	530/2075	45.1	107/237	14.1	185/1311
Substance‐related disorders	16.2	337/2075	10.1	24/237	21.2	278/1311
*Legal status*						
Voluntary admission	54.9	1140/2075	38.0	90/237	55.6	729/1311

a Marital status not stated/inadequately described for 13 not approached, 5 nonconsenters, and 12 consenters.

Logistic regression models adjusting for age, gender, Aboriginality, and marital status were used to examine whether primary psychiatric diagnosis was independently associated with risk status for each of the four behaviours, interest in changing each health risk behaviour, and acceptability of being provided advice and support for behaviour change (strongly agree/agree vs unsure, disagree, strongly disagree).

## Results

### Participants

**Figure 1:**
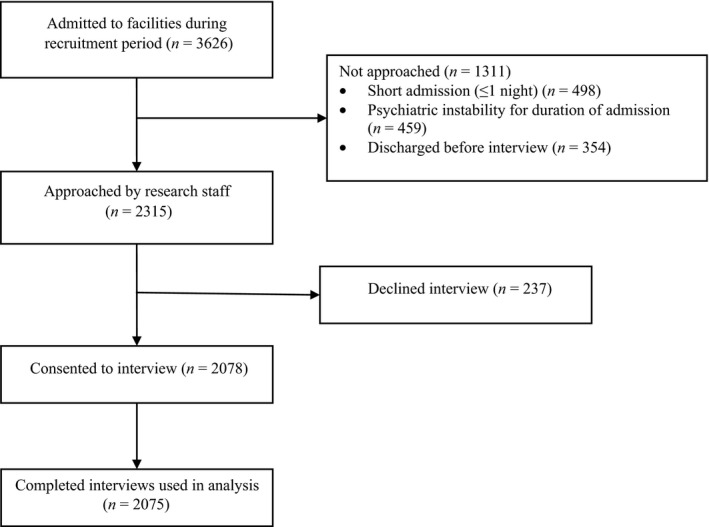
Participant flow diagram.

Of 3626 patients admitted to the four units during the study period, 2315 (63.8%) were approached (1311 not approached due to: short admission, *n *=* *498; psychiatric instability for duration of admission, *n *=* *459; or discharged before interview, *n *=* *354; Fig. 1). Of those approached, 2078 (89.8%) consented to participate. Three participants did not provide responses for any of the measures of this study and were excluded from analysis, providing a sample of 2075 participants and an overall completion rate of 57% (2075 of the 3626 patients admitted during the study period). Table 1 summarizes the participant characteristics and provides a demographic comparison of participants, nonconsenting patients, and patients not approached.

### Prevalence of health risk behaviours

At least 50% of participants self‐reported being at risk for each of the four behaviours (Table 2). Risk for inadequate nutrition was most prevalent (94.8%; inadequate fruit and inadequate vegetable consumption, 57.9% and 92.9%, respectively), followed by tobacco smoking (61.7%), hazardous alcohol consumption (50.9%), and inadequate physical activity (50.2%). Almost all participants (98.9%) engaged in at least one health risk behaviour (0 risks 1.1%; 1 risk 10.5%; 2 risks 34.8%; 3 risks 37.4%; 4 risks 16.2%), with 88.4% (1787/2021) of respondents at risk for multiple behaviours (two or more).

**Table 2:** Participants engaging in health risk behaviours and multiple risk behaviours, by primary diagnosis

Behaviour^a^	Total		Psychotic disorders		Mood disorders		Substance related		Personality disorders		Anxiety/stress related		Other	
	%	*n*	%	*n*	%	*n*	%	*n*	%	*n*	%	*n*	%	*n*
Smoking	**61.7**	**1277/2070**	64.1	339/529	48.9	319/653	80.7	271/336	56.1	162/289	69.5	130/187	73.7	56/76
Alcohol consumption	**50.9**	**1037/2037**	43.3	222/513	45.5	294/646	67.9	224/330	56.3	162/288	55.4	103/186	43.2	32/74
Overall nutrition	**94.8**	**1943/2049**	95.9	495/516	93.2	604/648	95.2	320/336	94.1	270/287	97.3	181/186	96.1	73/76
Inadequate Fruit	**57.9**	**1187/2050**	63.6	328/516	52.5	341/649	60.1	202/336	56.8	163/287	63.4	118/186	46.1	35/76
Inadequate vegetable	**92.9**	**1905/2051**	94.4	488/517	90.9	590/649	92.9	312/336	92.3	265/287	95.7	178/186	94.7	72/76
Inadequate physical activity	**50.2**	**1030/2050**	52.6	272/517	50.2	326/650	45.7	153/335	48.6	140/288	56.8	105/185	45.3	34/75

a Health behaviour risk status was defined in line with Australian national guidelines: any smoking (daily, weekly, less than weekly) (Ministerial Council on Drug Strategy 2004); consumption of more than two standard alcoholic drinks on a usual drinking day (National Health and Medical Research Council 2009); consuming less than two serves of fruit and/or less than five serves of vegetables per day (National Health and Medical Research Council 2003); or not engaging in at least 30 min of physical activity for at least 5 days a week (Department of Health and Aged Care 1999).

After adjusting for demographic variables, participants with substance‐related disorders (OR = 2.56), anxiety‐ or stress‐related disorders (OR = 1.51), or ‘other’ disorders (OR = 2.00) were most likely to be at risk for tobacco smoking, while those with mood disorders (OR = 0.53) or personality disorders (OR = 0.73) were least likely (Table 3). In regard to hazardous alcohol consumption, participants with substance‐related disorders were twice as likely to be at risk (OR = 2.00) while those with schizophrenia or psychotic disorders were least likely (OR = 0.62). No relationships emerged between diagnosis and risk of inadequate fruit and/or vegetable consumption or inadequate physical activity.

**Table 3:** Diagnostic associations with health risk behaviours

	Smoking			Alcohol			Fruit and/or vegetable			Physical activity		
	OR	95% CI	*P*	OR	95% CI	*P*	OR	95% CI	*P*	OR	95% CI	*P*
Psychotic disorders	1.07	0.86–1.34	.54	0.62	0.50–0.77	<.0001*	1.29	0.79–2.12	.31	1.20	0.98–1.48	.08
Mood disorders	0.53	0.43–0.65	<.0001*	0.85	0.70–1.04	.12	0.67	0.45–1.01	.06	0.92	0.76–1.12	.41
Substance related	2.56	1.90–3.46	<.0001*	2.00	1.54–2.59	<.0001*	1.02	0.59–1.78	.93	0.86	0.68–1.09	.22
Personality disorders	0.73	0.56–0.95	.02*	1.23	0.95–1.60	.12	0.88	0.51–1.52	.65	0.90	0.69–1.15	.39
Anxiety/stress related	1.51	1.07–2.13	.02*	1.25	0.91–1.71	.17	2.11	0.85–5.26	.11	1.34	0.98–1.82	.06
Other	2.00	1.14–3.47	.02*	0.72	0.44–1.18	.19	1.40	0.43–4.54	.57	0.81	0.51–1.29	.37

*p <* *.05.

Odds ratios are adjusted for age, gender, Aboriginality, and marital status.

### Interest in changing health risk behaviours and acceptability of being provided advice and support

The majority of participants who reported being at risk for a health behaviour indicated they were considering making changes to improve that behaviour (Table 4). Two‐thirds of smokers (65.3%) were seriously considering quitting or reducing their smoking, and 60.6% of those who reported inadequate physical activity were considering increasing their physical activity. Approximately half of those who were at risk for hazardous alcohol consumption or inadequate fruit and/or vegetable consumption were considering reducing their alcohol consumption (49.5%) or increasing their fruit and/or vegetable intake (47.6%). Eighty per cent of participants agreed or strongly agreed that it would be acceptable to receive advice and support from inpatient facility staff to improve these behaviours (Table 4).

**Table 4:** At‐risk participants interested in changing their health risk behaviours, and overall patient acceptability towards being provided staff advice and support, by primary diagnosis

Behaviour	Total		Psychotic disorders		Mood disorders		Substance related		Personality disorders		Anxiety/stress related		Other	
	%	*n*	%	*n*	%	*n*	*%*	*n*	%	*n*	%	*n*	%	*n*
Smoking	65.3	824/1261	62.1	205/330	68.0	215/316	65.9	178/270	67.5	108/160	62.3	81/130	67.3	37/55
Alcohol consumption	49.5	513/1037	41.9	93/222	50.0	147/294	60.7	136/224	42.6	69/162	52.4	54/103	43.8	14/32
Fruit and/or vegetables	47.6	924/1940	46.5	229/493	50.9	307/603	46.6	229/493	48.9	132/270	43.1	78/181	39.7	29/73
Inadequate physical activity	60.6	624/1030	57.0	155/272	62.9	205/326	64.7	99/153	60.7	85/140	58.1	61/105	55.9	19/34
*Acceptable to be provided staff advice and support*														
Strongly agree/agree	80.4	1630/2028	75.2	381/507	80.7	518/642	84.1	281/334	84.3	241/286	81.6	151/185	78.4	58/74
Unsure	6.5	131/2028	8.3	42/507	6.1	39/642	3.6	12/334	6.6	19/286	8.1	15/185	5.4	4/74
Disagree/strongly disagree	13.2	267/2028	16.6	84/507	13.2	85/642	12.3	41/334	9.1	26/286	10.3	19/185	16.2	12/74

Few differences in interest in improving health risk behaviours or acceptability of being provided such advice or support were found across diagnoses (Table 5). Participants with schizophrenia or psychotic disorders were least likely to be interested in reducing their alcohol consumption (OR = 0.65), while those with substance‐related disorders were most interested (OR = 1.78). Those with mood disorders expressed the greatest interest towards increasing their consumption of fruits and/or vegetables (OR = 1.24). Participants with diagnoses of schizophrenia or psychotic disorders were less likely to agree that it is acceptable to be provided advice and support from the inpatient facility staff to change their health behaviours (OR = 0.66).

**Table 5:** Diagnostic associations with interest in changing health risk behaviours and acceptability of being provided advice and support from inpatient facility staff

	Interest in improving health risk behaviours^b^												Acceptability		
	Smoking			Alcohol			Fruit and/or veg			Physical activity					
	OR	95% CI	*P*	OR	95% CI	*P*	OR	95% CI	*P*	OR	95% CI	*P*	OR	95% CI	*P*
Psychotic disorders^a^	0.83	0.63–1.08	.16	0.65	0.48–0.89	.006*	0.97	0.79–1.20	.77	0.86	0.64–1.15	.31	0.66	0.51–0.84	.0007*
Mood disorders^a^	1.17	0.89–1.55	.27	1.05	0.80–1.39	.72	1.24	1.02–1.52	.03*	1.19	0.89–1.57	.24	1.03	0.81–1.31	.81
Substance related^a^	1.04	0.78–1.39	.77	1.78	1.31–2.41	.0002*	0.91	0.71–1.17	.46	1.19	0.82–1.71	.36	1.36	0.99–1.88	.06
Personality disorders^a^	1.12	0.78–1.60	.53	0.74	0.52–1.05	.09	1.01	0.78–1.31	.94	0.93	0.64–1.35	.69	1.37	0.97–1.92	.08
Anxiety/stress related^a^	0.85	0.58–1.25	.41	1.13	0.75–1.70	.57	0.82	0.60–1.12	.20	0.90	0.60–1.37	.64	1.08	0.73–1.60	.69
Other^a^	1.09	0.61–1.95	.76	0.76	0.37–1.55	.45	0.73	0.45–1.19	.21	0.74	0.37–1.50	.41	0.88	0.50–1.54	.64

a Limited to those at risk for each behaviour; *p <* *.05.

b Odds ratios are adjusted for age, gender, Aboriginality, and marital status.

## Discussion

Psychiatric inpatients reported a high prevalence of risk for tobacco smoking, hazardous alcohol consumption, inadequate fruit and/or vegetable intake, and inadequate physical activity. For each of the four behaviours, at least half of the sample were found to be at risk. Almost all of the sample were at risk for at least one behaviour (98.9%), and the majority (88.4%) were at risk for two or more behaviours. The prevalence of some risk behaviours differed between diagnostic categories. Patients with substance‐, anxiety‐related, and other disorders were at an increased risk for smoking, and those with substance‐related disorders were most likely to consume alcohol at a hazardous level. For each risk behaviour, a half to two‐thirds of at‐risk participants expressed an interest in making changes to improve the behaviour. Participants with substance use disorders were most likely to be interested in reducing their hazardous alcohol consumption, whereas participants with schizophrenia or psychotic disorders were least likely to be interested in doing so. Participants with mood disorders were most likely to be considering increasing their fruit and/or vegetable consumption. The majority of participants reported that it would be acceptable for inpatient facility staff to provide them with advice and support to change their health risk behaviours during their inpatient stay. While those with schizophrenia or psychotic disorders were least likely to agree it would be acceptable, 75% of such patients nevertheless reported that it would be so. These findings highlight the need to develop strategies to improve the health risk behaviours of people accessing psychiatric inpatient care, and the need to identify strategies to elicit inpatients’ care expectations and to facilitate clinician understanding and response to such expectations.

The results are consistent with previous research, finding a high prevalence of health risk behaviours among patients of mental health services (Bartlem *et al*. 2015a; Dipasquale *et al*. 2013; Filia *et al*. 2011; Janney *et al*. 2014; Kilbourne *et al*. 2009; Kilian *et al*. 2006; Morgan *et al*. 2014; Prochaska *et al*. 2014). However, direct comparisons are difficult given differences in study setting, sample, and how health risk behaviours are defined. The majority of previous research has focused upon community or outpatient mental health services (Bartlem *et al*. 2015a; Dipasquale *et al*. 2013; Filia *et al*. 2011; Janney *et al*. 2014; Kilbourne *et al*. 2009; Morgan *et al*. 2014). Compared to a study undertaken among community mental health service clients in the same health district (Bartlem *et al*. 2015a), the current study identified a greater prevalence of at‐risk patients for each of the four health risk behaviours (smoking 62% vs 51%; hazardous alcohol consumption 51% vs 43%; inadequate fruit and/or vegetable consumption 95% vs 87%; inadequate physical activity 50% vs 47%). Moreover, the current study also identified that patients accessing inpatient psychiatric services may be more likely to engage in multiple health risk behaviours, with 88% at risk for two or more behaviours, compared to 78% among those accessing community mental health services (Bartlem *et al*. 2015a). This finding further emphasizes the need to address the long‐term disease health risk behaviours of people who access mental health services throughout contacts with all mental health service providers, by confirming a similar if not greater need exists in psychiatric inpatient facilities. Further, the high prevalence of risk across all diagnostic groups suggests that addressing physical health concerns should be a priority among all patients of mental health facilities, in contrast to a narrow focus on those with severe mental illness.

The finding that a large proportion of at‐risk participants were considering making positive changes to health risk behaviours and that the large majority would find it acceptable to be provided advice and support during their inpatient stay is consistent with previous research (Bartlem *et al*. 2015a; Buhagiar *et al*. 2011; Filia *et al*. 2011; Ussher *et al*. 2007). The level of interest identified for increasing fruit and vegetable consumption and physical activity, and reducing alcohol consumption, is similar to that found in a study of US psychiatric inpatients (Prochaska *et al*. 2014). Interest in change in the current study, and acceptability towards being provided support from their psychiatric inpatient facility were comparable to the interest identified among clients of community mental health services in the same local health district in which this study was conducted (Bartlem *et al*. 2015a,b). This finding strengthens the argument for enhancing the capacity of mental health inpatient services to include health risk behaviour as part of treatment interventions.

During the inpatient stay, physical health, including modifiable health risk behaviours, should be assessed and recorded to enable appropriate follow‐up (Glasper 2016; Lambert *et al*. 2017). This could include brief advice or counselling, or engagement in healthy lifestyle interventions (Baker *et al*. 2011; Harrison *et al*. 2012; Lambert *et al*. 2017). At a minimum, it should include referral to appropriate physical health and behaviour change supports, and the development of a physical care plan upon discharge that is communicated to relevant services to enable continuation of care, including community mental health and primary care providers (Baker *et al*. 2011; Harrison *et al*. 2012; Lambert *et al*. 2017; Royal College of Physicians & Royal College of Psychiatrists 2013). Brief physical health assessment tools such as the Health Improvement Profile (White *et al*. 2009) have been trialled successfully in community‐based mental health (Bressington *et al*. 2014, 2017) and primary care settings (Hardy *et al*. 2014). Preliminary evaluations have shown that use of such a tool is acceptable to both consumers and care providers (Bressington *et al*. 2017) and can lead to improvements in consumer health risk behaviours (Bressington *et al*. 2014). To the authors’ knowledge, such physical health assessment tools or brief programs are yet to be evaluated in the inpatient setting. There is a need to explore the feasibility and acceptability of providing physical health assessments and brief programs within the inpatient setting and to explore the strategies that may be required to support mental health clinicians to provide such care.

The variation by diagnostic category in interest in improving health behaviours and perceived acceptability of receiving support from mental health clinicians to do so adds to a very small body of research. Two studies in the United Kingdom have found no differences in interest in improving physical activity (Buhagiar *et al*. 2011; Ussher *et al*. 2007), smoking, and diet (Buhagiar *et al*. 2011) across psychiatric diagnostic groups, while an Australian study found those with depression had increased interest in quitting smoking and in increasing physical activity (Bartlem *et al*. 2015a). The findings across all diagnostic groups of a high interest in improving risk behaviours, and high acceptability towards receiving advice and support during an inpatient stay provide further evidence of the need to address these behaviours for all people with a mental illness.

To the authors’ knowledge, this study is the first to examine whether psychiatric inpatients would find it acceptable to be provided advice and support from inpatient facility staff for improving a range of health risk behaviours. The findings add to a growing body of research in other settings indicating that people with a mental illness find acceptable and hold an expectation of being provided such care, and are interested in improving their health risk behaviours (Bartlem *et al*. 2015a,b; Buhagiar *et al*. 2011; Filia *et al*. 2011; Prochaska *et al*. 2014; Ussher *et al*. 2007). Given that various studies have suggested that some mental health clinicians hold a dissonant view that their patients have neither an interest in, nor find such care provision to be acceptable (Chwastiak *et al*. 2013; Johnson & Fry 2013; Johnson *et al*. 2009; Robson *et al*. 2013), further research is required regarding the mechanisms to modify such views as a means of increasing such care provision.

### Limitations

This study includes a large and diverse sample of psychiatric inpatients and a high consent rate, with 90% of those approached consenting to the study. The authors have speculated reasons for the high consent rate, and it is possible that the nature of the inpatient setting contributed to patients being more amenable to engage in conversation with research staff in the units. The findings should be interpreted in the light of its methodological characteristics. Firstly, use of patient report of health behaviours introduces possible recall and/or social desirability response biases, which may have led to conservative estimates of the prevalence of health risk behaviours (de Beaurepaire *et al*. 2007). Similarly, social desirability of response may have contributed to an overestimate of reported interest in changing these behaviours and in acceptability towards being provided with preventive care. Secondly, the analysis of associations between psychiatric diagnosis with risk behaviour prevalence, interest towards changing behaviours, and acceptability of being provided advice and support included primary psychiatric diagnoses only. It is possible that accounting for the presence of co‐occurring diagnoses may have impacted on the results of the association analyses. Those participants whose primary diagnosis was substance related may have been admitted with an alcohol use disorder, which may have confounded the association between this diagnostic group and hazardous alcohol consumption outcomes. Finally, given the study was conducted in a single health district in one state in Australia, the generalizability of the findings to other jurisdictions and settings is unknown.

## Conclusion

The current study adds to the limited data regarding the prevalence of health risk behaviours and interest in making positive changes to improve health risk behaviours among psychiatric inpatients. It further demonstrates patient acceptability towards being provided support and advice for these behaviours in a psychiatric inpatient service setting. The results attest to the need to address the high prevalence of health risk behaviours among people with a mental illness and to address the barriers to clinician provision of care as a means of doing so.

## Relevance for clinical practice

The high levels of behavioural risks, interest in improving such risks and acceptability of being provided with support and advice for these behaviours attest to the need for mental health clinicians working in inpatient psychiatric settings to routinely provide preventive care to address behaviour change. The high prevalence of risk, interest and acceptability across all diagnostic groups further suggests that clinicians should address behavioural risks among all patients, regardless of psychiatric diagnosis. There is a need to enhance the capacity of mental health inpatient services to address health risk behaviours as part of routine care. During an inpatient stay, this should include assessment and recording of any health risk behaviours. At a minimum, follow‐up to such assessment should include the provision of brief advice and referral to behaviour change supports. Additional care should include brief counselling and the development of a physical care plan to ensure ongoing communication with services providing care postdischarge. There is a need to identify feasible ways in which inpatient services can systematically provide such care, and services need to provide clear guidance to clinical staff regarding processes for identifying risk and providing follow‐up behaviour change support. Further, mental health services need to explore effective strategies for supporting inpatient mental health staff to provide such care, including the provision of organizational supports and systems change.
